# Appropriateness in Dentistry: A Survey Discovers Improper Procedures in Oral Medicine and Surgery

**DOI:** 10.1155/2018/3245324

**Published:** 2018-04-04

**Authors:** Giacomo Oteri, Vera Panzarella, Antonia Marcianò, Olga Di Fede, Laura Maniscalco, Matteo Peditto, Giuseppina Campisi

**Affiliations:** ^1^Department of Biomedical and Dental Sciences and Morphofunctional Imaging, University of Messina, via Consolare Valeria University Hospital “Gaetano Martino”, 98124 Messina, Italy; ^2^Department of Sensorineural and Movement Surgery, Oral Medicine and Dentistry for Patients with Special Needs, University of Palermo, via del Vespro 129, 90127 Palermo, Italy; ^3^Department of Surgical, Oncological and Oral Sciences, University of Palermo, via Liborio Giuffrè 5, 90123 Palermo, Italy

## Abstract

**Objectives:**

The aim of this study was to assess appropriateness of diagnostic exams, treatments, and procedures among Italian dental practitioners.

**Materials and Methods:**

A questionnaire with multiple responses on topics of dentistry and oral medicine was administered to a sample of 198 Italian dental practitioners. Information on characteristics of the respondents was also collected. Descriptive statistics and multiple correspondence analysis (MCA) were applied. Data were analyzed using R software (version 3.3.2).

**Results:**

The survey respondents included Doctors of Medicine (MD) (54/198 = 27%) with or without specialty in dentistry (33% versus 67%), Doctors of Dental Surgery (DDS) (144/198 = 73%), DDS with specialty in orthodontics (7%), and DDS with specialty in oral surgery (4%). Mandatory procedures in dental and oral medicine education and training include (a) prescription of antibiotics before/after oral surgery procedures; (b) prevention strategies for oral cancer, and (c) prescription of dental X-ray examinations (41%, 52%, and 48%, resp.).

**Conclusion:**

On examining the results of the survey, it is evident that information and implementation of the above mentioned procedures are crucially needed. Our results confirm the necessity to reduce inappropriate practices in dentistry, implementing formation and information, leading to correct prescriptions, and optimizing patients' oral health. This coincides with the Italian Slow Medicine program entitled “*Fare di più non significa fare meglio–Choosing Wisely Italy*,” which has also motivated this study.

## 1. Introduction

Over the last decades, the concept of appropriateness of health-care procedures has been attracting considerable attention. Several studies clearly state that escalating health-care costs and identification of inappropriate care have led to a critical examination of possible overuse and underuse of many medical and surgical procedures and questions as to when or whether they are needed [1]. These issues can influence government decisions and healthcare plans. In this regard, the recent setting out of eligibility conditions and prescriptive suitability guidelines available under the Italian National Healthcare System has created strong perplexities, due to the financial impact on the economic and public health-care fields of inappropriate drug prescriptions and medical procedures [2].

Among nonprofit organizations, the Slow Medicine board of directors, which since its foundation has dealt with the issue of clinical appropriateness, immediately expressed many doubts regarding the performance of healthcare professionals who are called upon to apply it and to discriminate between rationing and appropriateness [3]. The issue of prescriptive appropriateness should have the purpose to limit to strictly necessary medical exams and procedures but not only from a merely economic point of view but rather in the light o a patient-centered orientation. On the other hand, government's rules are proposed as guidelines but appear to have the sole-stated objective of reducing costs with threat of sanctions for doctors who do not respect them and moreover hardly applicable, represents an implicit rationing. Defining proper guidelines for any given procedure appears, at this point, to be of crucial importance. It must be inspired to the best scientific knowledge coming from evidence in the most updated literature in order to trace the right diagnostic and therapeutic path. The World Health Organization (WHO) estimated the economic burden of ineffective care, accounting for between 20% and 40% of healthcare expenditure [4]. In 2010, the American Board of Internal Medicine (ABIM) Foundation launched a project “Choosing Wisely” [5]. The project aims to reduce inadequate medical practices to ensure more accurate diagnoses and avoid the waste of limited resources. Slow Medicine, an Italian movement based on the principle that quantity does not imply quality, is perfectly in line with the aims of Choosing Wisely, promoting the concept that some procedures may not only not be beneficial to patients but could even turn out to be harmful [5]. In December 2014, the Sicilian Healthcare Department started a project to verify the quality of healthcare services in the Sicilian Region (*Progetti Obiettivo* (*PSN 2013*)*—Azione 16.2*). This project involved “Papardo” Hospital, Messina, Italy, in collaboration with the Universities of Messina and Palermo (Sicily, Italy), and falls into the sphere of the Slow Medicine “*Fare di più non significa fare meglio–Choosing Wisely Italy*” initiative. It is intended to promote adequate healthcare practices amongst physicians and other healthcare professionals [6]. The Italian Federation of Surgeons and Dentists [7] has recently joined the project. There are a number of procedures in oral medicine and oral surgery in which the problem of appropriateness has a wide impact in the field of public health. Just think about the indiscriminate prescription of antibiotic, in which the dentist certainly has a preponderant role: the use of not always necessary radiodiagnostic exams or the delay in the diagnosis of oral carcinoma for which numerous campaigns of sensitization have been conducted in Italy as in other countries. Other papers analyze the competencies of dental practitioner in a different point of view. An example could be the study of Field et al. [8] that consider methods of teaching, learning, and assessment that help to overcome some of the more traditional barriers within dental undergraduate programmes, considering four domains (e.g., Professionalism, Safe and Effective Clinical Practice, Patient-Centred Care, and Dentistry in Society) [8]. The aim of this survey was to assess, in a defined number of Italian dental practitioners, the clinical appropriateness of the most frequently prescribed diagnostic exams, pharmacological treatments, and procedures. The study was considered an exploratory trial, having the attempt to proof the feasibility and acceptability of a questionnaire which might have the potential to raise awareness on appropriateness in dentistry procedures. The initiative inspired to the Slow Medicine campaign arises from the need to give a guidance for appropriate prescription criteria in the management of the care of patients from a strictly clinical point of view. Therefore, this study is to be considered a preliminary study that will be implemented repeating the survey on national scale to delineate a portrait of the Italian situation. We hope that as promoted by Slow Medicine, other countries will strive to identify the clinical appropriateness of the most frequently prescribed diagnostic exams, pharmacological treatments, and procedures in dentistry. Since the sample is not probabilistic and, consequently, not representative of the Italian dentist population, the obtained results can be seen as preliminary. These results can be useful for further investigations on this topic.

## 2. Materials and Methods

**Figure 1:**
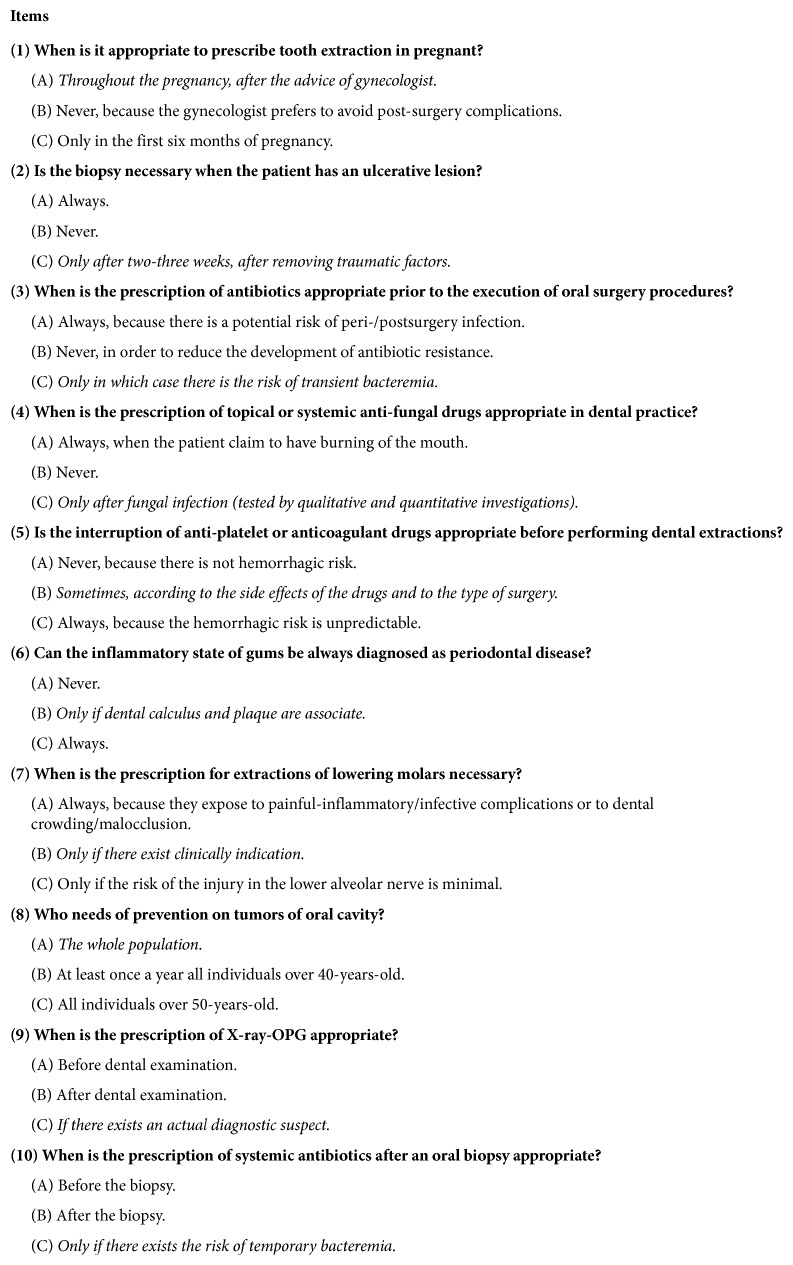
Sample of the administered survey (Items 1–10) with the correct answer underlined.

During a series of conferences and symposiums held in Sicily, Italy, between November and December 2016 on Dental and Oral Health, promoted by the National Association of Italian Dentists (ANDI) and the *Continuing Education in Medicine* (ECM) program, 291 participants were given a questionnaire during registration. All the participants were invited to participate in the survey. Subjects demonstrated their consent by delivering the form to the dedicated desk, at the end of the conference. This questionnaire, not sponsored by any company, was anonymous and voluntarily answered by professionals. It was drawn by a panel of 5 experienced oral surgeons and 5 oral pathologists, suggested by the Italian Society of Oral Surgeons (SIdCO) and the Italian Society of Oral Pathology and Medicine, (SIPMO) that approved its content, prior the administration to the professionals. The questionnaire included demographic variables of the responding practitioners, such as age, gender, degree (“Dentistry and Prosthetics” or “Medicine and Surgery”), postgraduate qualifications (None, Dentistry, Orthodontics, and Oral Surgery), and professional qualifications (Public, Private, Public, and private). The classification of dental practitioners is in line with Italian ordination that consists of three qualified figures for exercise the dental practice: general practitioner, specialist in orthodontics, and specialist in oral surgery, all respondents were participants of a scientific event within the continuing education in medicine program. Knowledge variables included questions on oral surgical procedures (5 items) and oral medicine and pathology (5 items). Multiple-choice questions, each with three answers, of which only one was correct, were submitted to participants, on the above mentioned 10 items, which are specified in Figure 1. Finally, a quantitative variable, “number of errors,” was created.

### 2.1. Statistical Analysis

Descriptive statistics were carried out. Specifically, continuous variables were summarized with mean and standard deviation, whereas categorical variables were summarized with frequency distributions. The associations between variables were assessed with Chi-square and Fisher's exact test. Additionally, logistic regression model was applied for each item of the questionnaire. Eventually, multiple correspondence analysis were carried out on the collected data and *p* value was set to <0.05 as significant. All data were analyzed using R software version 3.3.2.

## 3. Results

**Figure 2:**
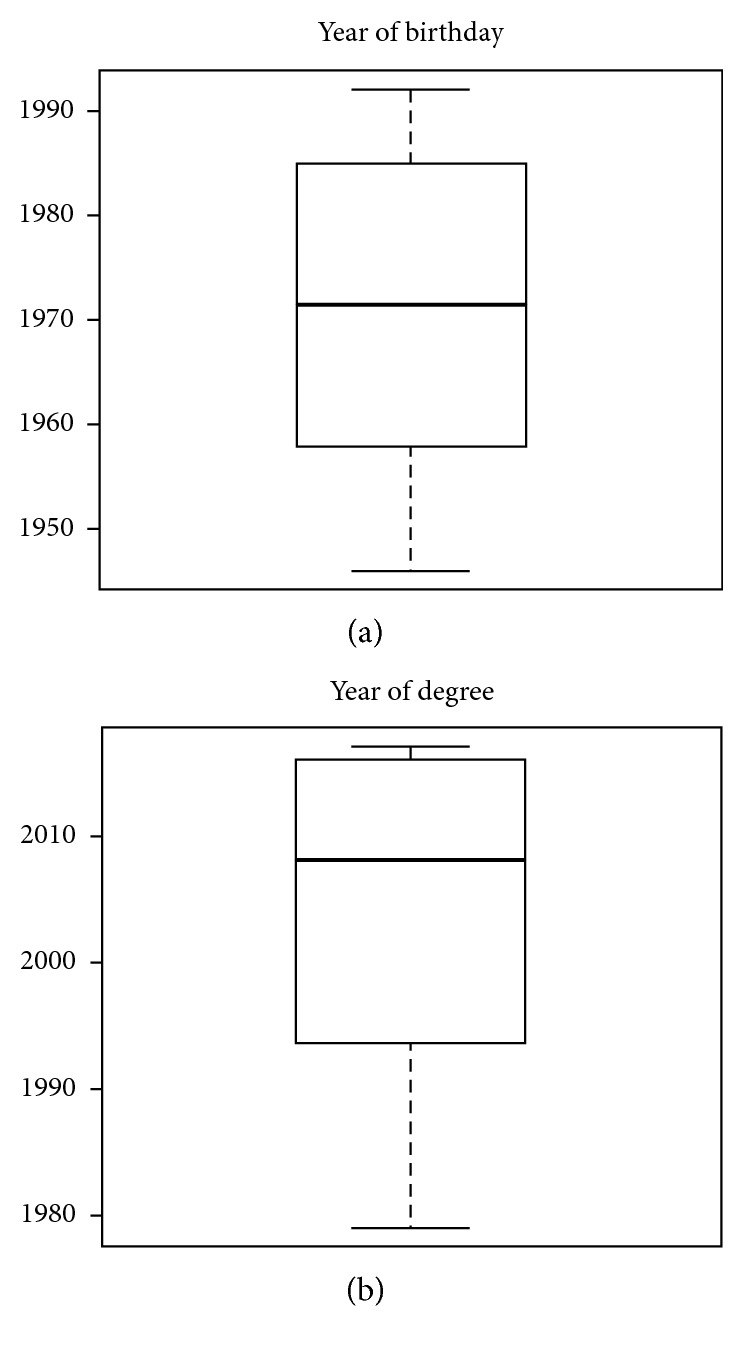
Boxplots of (a) year of birthday and (b) year of degree that shows a symmetric and an asymmetric distribution of the two variables, respectively.

**Figure 3:**
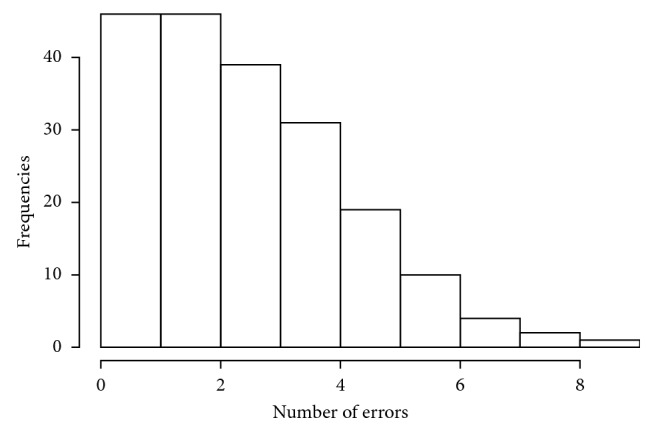
Histogram of number of errors that exhibits an asymmetric distribution.

**Figure 4:**
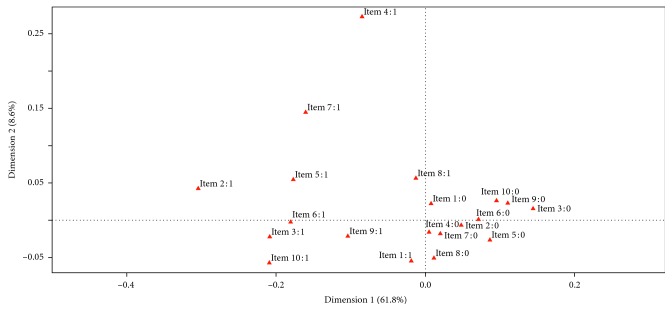
Graph of multiple correspondence analysis that highlights a homogeneity in the answer.

The questionnaire was answered by 198 dental practitioners, who represented 70.4% of all participants. 63% of respondents were male (mean age 46.36 (SD = 13.54)), and 37% were female (mean age 40.59 (SD = 12.94)). 27% graduated in Medicine and Surgery while the remaining 73% had a degree in Dentistry and Dental prostheses. About 78% of the subjects worked in both public and private structures, 21% in private structures, and only 1% in public structures (Table 1). Boxplots of variables regarding year of birth and year of degree (Figure 2) show a symmetric distribution of year of birth ranging from 1946 to 1992. Distribution by year of degree, instead, shows a negative asymmetric distribution, ranging from 1979 to 2017, indicating a high percentage of older respondents. With regard to qualifications (Table 2), 33% (M/F = 17/1) of MDs were specialized in Dentistry versus 67% (M/F = 26/10) who did not have any specialization. Among DDS, 89% (M/F = 74/54) did not have any specialization, whereas 7% (M/F = 4/7) and 4% (M/F = 3/3) had a specialization in orthodontics and oral surgery, respectively. To analyze the answers given in the questionnaire, the variable “number of errors” was created. This variable had a value of “0” for each correct answer and value of “1” for each incorrect answer, recommending an unnecessary procedure/prescription. The distribution of this variable, obtained from the total number of incorrect answers (level “1”) per question for each subject, had a positive asymmetric distribution, with a high concentration of a minimum number of wrong answers marked by each participant (Figure 3). This means that only a few respondents gave more than four wrong answers.  Table 3 shows the percentages of correct and incorrect answers given by the subjects on the 10 questions (valued as “0” or “1”). It can be seen that the questions with fewer errors, numbers 2, 4, and 7, are related to biopsy in the case of ulcerative lesions (86% of correct answers), prescription of topical or systemic antifungal medication (94%), and tooth extraction of the lower molars (88%). Larger percentages of errors were found regarding questions 3, 8, and 9, with 41%, 48%, and 52%, respectively. These concern the administration of antibiotics prior to the execution of an oral surgery procedure, prevention of oral cavity tumors, and prescription of the X-ray-OPG (orthopantomogram) diagnostic examination.  Table 4 shows the percentage distribution of frequency of the number of errors correlated to specialization. Questions 2 and 7, related to a biopsy when a patient has an ulcerative lesion and the prescription of tooth extraction of lower molars, had the highest percentage of correct answers. For these questions, the best scores were obtained by those specialized in oral surgery (DDS). The best score for question 4, regarding the prescription of topical or systemic antifungal drugs, was obtained by those who do not possess any specialization. Questions 8 and 3 are related to prevention against the development of oral cancer and prescription of antibiotics prior to the execution of oral surgery procedure, respectively. The worst scores for both were obtained from orthodontic specialist answers. Question 9 is related to the X-ray-OPG diagnostic test. The worst score was obtained by dental specialists. The chart in Figure 4 shows the multiple correspondence analysis (MCA). The main objective of MCA is to analyze existing relationships between a set of qualitative variables by reducing the size. This reduced dimension can reproduce most of the association between the variables analyzed in a small number of factors. The horizontal axis represents the first dimension and explains the 61.8% while the vertical axis explains the 8.6%. It can be seen that subjects who make a mistake in a question are more likely to give a wrong answer to the other questions and vice-versa. The axes are useful to highlight homogeneity in the questions. The *y*-axis distinguishes erroneous answers to questions 4 and 7 that show a considerable distance from the other elements, indicating that there is more knowledge that can be translated into good prescription of topical or systemic antifungal medication and in extractive surgery of dental extraction. Additionally, there is good appropriateness in the prescription of treatment of ulcerative lesions, while there is a lack of adequacy regarding questions on antibiotics, identified in questions 3 and 10. Logistic regression models for items 1,6,7 and 9 revealed significant differences (*p* = 0.01) in relation to dentistry degree, years of birth, intercept and professional activity (private and public) (Table 5).

## 4. Discussion

The purpose of the study was to discuss about the clinical appropriateness of some drug/exam prescriptions and preventive/diagnostic measures tested among a defined group of Italian dental practitioners in view of the recent update of national guidelines issued by the Ministry of Health, promoting oral health and preventing oral diseases [9]. To our knowledge, this is the first Italian survey involving dental practitioners on appropriateness in dentistry procedures. To pilot the survey, we chose a sample of Italian dental practitioners of similar age and background who attended the scientific events within the Continuing Medical Education program due by state. The appropriate sample size to answer the pilot survey was determined doing an average between participants to national and international conferences whose proportion usually for Italy is 1 : 10. This pilot survey conducted on a regional scale appeared to be less time-consuming and resource-consuming to pretest the survey and eventually highlight errors in the administration procedure and/or comprehension difficulties. The proposed study should be conducted in other countries, in order to analyze the differences on a national level before being administered in an international conference. Therefore, the results must be analyzed according to the limited dimension of the sample. Moreover, as a general limit, the autoselection of participants to the pilot study (dentists that willingly participates in Congresses or Conferences) confirms that the group is not statistically representative of a general cohort of dentists and could represent a starting point for more extensive investigations. 63% of respondents were male, and 37% were female. This result is in contrast with the current situation which sees a greater number of women enrolled in the degree course of Dentistry [10]. From a sociological perspective, it could be an interesting aspect of the presence of women at conferences. It could be suggested that a woman less likely to participate to training events generally held at weekends. Only 1% percent of physicians work in public structures, while 21% work in private practice, and there is a 78% percent working both in a public and in a private structure. This could be explained in view of the average income of dentists working in private practice compared to the average income of hospital dentists. The Chi-square and Fisher's exact tests, used for this dataset, highlighted the presence of marginal association between sex and degree and sex and specialty (*p* value < 0.01), and between degree and specialty (*p* value < 0.0001). Eventually, there is a marginal border-line association between specialty and professional activity (*p* value = 0.049). There is not further marginal association between the other categorical variables. The explanatory variables used in all the models are sex, degree, specialty, and professional activities (where the “public” category was excluded due to the low frequency) and to identify the age of the respondent, it was create a binary variable obtained considering the median of the variable years of birth that was properly dichotomized in “≤1972” and “>1972”. We applied a logistic regression model for each items of the questionnaire in Figure 1. The items are all dichotomous and their categories are “0” that indicates the right answer, whereas “1” indicates the mistake. The coefficients of the logistic regressions give the change in the log odds of the outcome for a one unit increase in the predictor variable, in case of a quantitative variable or an increase when we change categories of a qualitative variable. The reduced model showed in Table 3 has been obtained after a stepwise model that compares the models with Akaike Information Criterion. In the first model, that is related to Item 1, only the coefficient associated with degree variable is statistically significant (*p* value = 0.007). This item regards the prescription of tooth extraction in pregnant. Since the baseline includes medicine and surgery degree, the probability to commit an error in Item 1 if the respondent has a dentistry degree changes the log odds of make mistake by 1.50. So it is 4.50 times more probable to commit an error in Item 1 if the respondent has a degree in dentistry with respect to the baseline (medicine and surgery degree). Whereas, the coefficient associated with gender shows a *p* value borderline (*p* value = 0.08). So there is, for the first item, more competence of the subjects with degree in medicine and surgery with respect to the subjects with degree in dentistry. In fact, the conditional probability to make an error on Item 1 for subjects with degree dentistry is equal to 91%. The models associated to Items 6 and 7, that are related to the inflammatory state of gums and prescription of the extraction of the lowering molars, respectively, show that only the coefficients of years of birth are significant for both of them. Since this is a dummy variable, being born after 1972 reduces the log odds by −0.82. Whereas, for Item 7, being born after 1972 increase the log odds by 1.39. In terms of the odds ratio is 0.44 and 4.04 times more probable make a mistake if the respondent is born after 1972 in Items 6 and 7, respectively, with respect to being born before 1972. In relation to Items 6 and 7, respondents born before 1972 resulted to be more competent than respondents born after 1972. Eventually, the Item 9, associated with the prescription of X-ray-OPG, has only the professional activity's coefficient statistically significant. This indicates that having a private and public professional activity decreases the log odds by −0.96. The odds ratio associated with this coefficient shows 0.38 times more probable to make mistake in Item 9 if the professional activity is public and private instead of having only a private activity. In all the other models applied for the items, there are no statistically significant coefficients. The subjects who have private and public professional activity are less competent in Item 9 with respect to the subjects who have only a private activity. Based on the results of the survey, the topics on which to raise awareness are mainly related to (i) prescription of antibiotics before/after oral surgery procedures; (ii) prevention strategies for oral cancer, and (iii) prescription of dental X-ray-OPG in dental practice. Regarding the first topic, notably frequent incorrect replies were given to the question “When is the prescription of antibiotics appropriate prior to the execution of oral surgery procedures?” (question 3). 59% of dental practitioners gave the correct answer (C). Only in cases where there is the risk of transient bacteremia. In particular, 39% of participants answered that antibiotic therapy is always appropriate because of the potential risk of peri-/postsurgery infection (answer A), and the remaining 2% would never prescribe antibiotics prior to the execution of oral surgery procedures in order to limit the development of antibiotic resistance (answer B). In relation to specialization, the worst scores were obtained by orthodontics (60%) and dental practitioners without specialization (41%). According to the most recent European Centre for Disease Prevention and Control (ECDC) report, Italy is one of the countries with the highest prescription rates. Despite a small, not significant decrease in the consumption trend for 2010–2014, Italy showed a population-weighted mean value higher than EU/EAA mean, second only to France [11]. Despite numerous Italian Medicine Agency (Agenzia Italiana del Farmaco–AIFA) communication campaigns, inappropriate antimicrobial prescription is a quality-of-care issue, especially since it is used for generic and inappropriate prophylaxis [12]. The improper prescription of antibiotics can lead to antimicrobial resistance, considered by WHO as one of the biggest threats to global health, since this particular misuse of antibiotic drugs leads to longer hospitalizations, higher medical costs, and increased mortality [13]. According to a recent literature review, antimicrobial prophylaxis (AP) is prescribed in many oral and dental treatments without any scientific background to support it [14]. For general dental practitioners, the most worrying aspect is related to infective endocarditis (IE) and to the risk of implant infection for patients with hip and knee prosthetic joints. AP is also prescribed in third molar surgery to reduce the incidence of postsurgical infections and to relieve complications such as swelling and pain and in dental implant placement surgery to prevent peri-implant infections and implant failure [14–17]. AP is usually prescribed by GDPs to prevent local infections (i.e., localized osteitis and soft tissue infection) at surgical sites and to avoid postoperative complications (i.e., pain, wound breakdown, impaired healing, necrotic bone exposition, soft tissue swelling, trismus, localized/generalized lymphadenopathy, erythema, intra-/extraoral sinus, and implant/flap failure) [14]. It is intended for healthy patients extending the prescription throughout the postsurgical period (from 6–8 hours to 5 days after surgical procedures) [18]. There may be several reasons for these abuses of AP, ranging from defensive medicine, social factors such as patient's expectation and demand, up to the unfounded belief of covering either a defect in aseptic clinical technique or improperly sterilized equipment, but one, above all, is the practice of the totally aleatory just-in-case principle [19]. With regard to AP and prevention of systemic infections, a review of literature shows that the most common use of AP in dentistry is generally intended to prevent IE and prosthetic joint infection (PJI) [18]. The evidence for AP efficacy to prevent PJI after dental treatments is scanty given that no randomized controlled trials have been carried out [18]. According to the guidelines elaborated by the American Dental Association (ADA) together with the American Academy of Orthopedic Surgeons (AAOS), AP should be considered only for patients with total joint prostheses within the first two years after replacement and/or in the presence of comorbidities [20]. Moreover, recent reports have focused on the issue of AP for IE prevention. According to the 2015 National Institute for health and Care Excellence (NICE) clinical guidelines (with 2016 amendment), “AP against IE is not recommended routinely for people undergoing dental (or other) procedures” [21]. Other guideline committees around the world recommend AP for high-risk individuals undergoing high-risk invasive dental procedures but the definition of “high-risk patient” is not universal, being considered different in the USA, in Europe and in Africa. Consequently, local (national) guidelines are strongly recommended, and, once established, information to dentists should be clearly described [14]. Very recently, data have shown that AP was highly cost effective compared to the cost of treating IE, and to date, there are no data concerning the risk of inducing antibiotic resistance associated with AP for IE prevention. Thus, generally, the worldwide stance adopted, limiting AP use to a restricted number of individuals and procedures, seems more appropriate, but more focused and better-powered studies should be conducted [20]. It follows that the precautionary antibiotic prescription principle, to date very often applied indiscriminately to all patients before any oral surgery procedure to prevent IE, should never be a basis for an appropriate prescriptive approach. The evidence of actual confusion among dental practitioners regarding the issue has made it necessary to spread key recommendations at the local level to clarify this critical point. The second most frequent mistake was related to the question on cancer prevention: “Who needs prevention of tumor of oral cavity?” (Item 8). Possible replies (and percentage of respondents) were (A) “The whole population” (53%), (B) “At least once a year all individuals over 40-years-old” (31%), and (C) “All individuals over 50-years-old” (16%). Almost half the respondents answered incorrectly (answer B and C), likely thinking only about early diagnosis (secondary prevention). Oral cancer accounts for around 6–10% of malignant diseases (10% in men and 4% in women) in the world. The most common form of oral cancer is squamous cell carcinoma (80%), followed by adenocarcinomas of salivary glands (20%) and other bone/connective tumors, (e.g., lymphomas and sarcomas). In Italy, the mean incidence of oral squamous cell carcinoma (OSCC) is relatively common: in 2012, registered incidences were 4.1 for male and 2.1 for female, with an age-standardized rate per 100,000 individuals per year [22]. Although the highest incidence of oral cancer is observed between the ages of 50 and 70 years, an increasing trend among young people has been recorded; therefore, the entire population should be subject to appropriate preventive protocols [23]. Survival after a case of OSCC is linked to stage of tumor at diagnosis. The majority of cases are identified late and in an advanced clinical stage (i.e., III or IV). Although OSCC is almost always preceded by visible and symptomatic early changes of the oral mucosa (such as ulcer, erithroplakia, leukoplakia, bleeding, and pain), general practitioners and dental professionals tend to underestimate them [24]. As a consequence, self-medications and/or inappropriate medications are carried out, in the false opinion of improving the course of the disease, while substantially increasing the duration of diagnostic delay [24]. Moreover, after primary treatment, relapses or metastases have been found in more than half of patients (80% of cases within the first two years), while the 5-year survival rate is, overall, less than 50% and quality of life significantly compromised by surgical and supportive therapies [25]. Primary and secondary prevention strategies are crucial and should be started in the second and third decades of a patient's life. However, since an uncontrollable portion of the diagnostic delay is represented by “patient delay” (defined as “the period between the patient first noticing symptoms and their first consultation with a health-care professional concerning those symptoms”) responsibility for the delay cannot be laid at the door of the treating healthcare professional [25]. Consequently, the whole population (not only people at risk of developing an oral cavity neoplasm) should be subjected to primary prevention, to monitor risk habits and, above all, to provide adequate information on oral cancer and preventive strategies. The need to produce, spread, and update recommendations for clinical practice comes also from recent scientific evidence [26]. In a large multicentric Italian study, patients' knowledge of oral cancer appeared to be high; overall, however, it did not appear that this information was being provided by clinicians: the majority of patients do not receive counseling on oral cancer knowledge and prevention from their physicians or dentists [26]. Therefore, patients with a history of unhealthy lifestyles such as smoking and excessive consumption of alcohol, and among younger subjects those with a history of HPV infection, should be subjected to a more accurate inspection of the oral cavity [27]. There are no proven, effective screening tests in oral cavity carcinoma, and there is also a lack of diagnostic criteria [28]. The role of adjunctive techniques that may facilitate early detection of oral premalignant and malignant lesions has been debated. Clinicians who use these visualization adjunctive tools may be unaware of the state of the evidence supporting their effectiveness [29]. Results obtained from the present survey support this inappropriate clinical management and suggest that clinicians need to improve their knowledge regarding prevention and early detection strategies. Educational interventions and oral health promoting events (i.e., Oral Cancer Day) supported by Universities and/or Scientific associations are necessary to increase professional awareness and to emphasize the key role of clinicians in prevention of oral cancer [30]. The third most common incorrect answer regarded the question on prescription of dental X-ray-OPG (Item 9: “When is the prescription of X-ray-OPG appropriate?”): 52% of respondents replied incorrectly (answers A and B). In particular, 44% of the participants answered that they usually prescribe X-ray-OPG after an initial visit rather than if there is a valid diagnostic issue (answer C, which was the correct one). The dentist is mainly responsible for OPG prescriptions and their overall frequency. A clinical examination is mandatorily performed before an OPG, which cannot be considered as a screening tool [31]. The behavior of Sicilian dental practitioners regarding X-ray-OPG prescription is in line with other European and North American countries where unfortunately a nonappropriate use of dental radiographs is observed. In the United Kingdom, routine screening by panoramic radiography is a common practice, with 42% of dental practitioners prescribing it despite having no clinical indication supporting this kind of radiographic examination [32]. More than half of the patients screened this way receive no benefit whatsoever from the panoramic films. To provide a proper dental X-ray prescription, the clinician must follow the 1977 Recommendations of the International Commission on Radiological Protection, later known as justification, optimization, and dose limits [33]. To justify the radiological diagnostic procedure, both the radiological medical practitioner and the prescribing clinician must provide the patient with a benefit, in terms of diagnostic information and potential therapeutic results, which exceeds the detriment caused by the examination [33]. A dental X-ray diagnostic procedure is optimized whenever the best possible balance between image quality and patient dose is achieved, following the “as low as reasonably achievable” (ALARA) principle [34]. As per European Union, referring practitioners must follow Euratom Directives (ref: c, d) and ICRP guidelines in order to avoid Unnecessary Medical Exposure [35]. In the present survey, among the issues with the highest response rate, a high level of knowledge was seen in the field of biopsies, prescription of antifungal medications and of indications for tooth extraction of the lower molars. Regarding question 2 (“Is a biopsy necessary when the patient has an ulcerative lesion?”), 86% of the respondents, mostly oral surgeons, showed that they were aware of what the right behavior is within the field of biopsies on oral ulcers, carrying out this surgical diagnostic procedure 2-3 weeks after removing any injury (answer C). Under the “prudence concept,” 11% of the respondents would always do a biopsy in the case of ulcerative lesion in the oral cavity (answer A) and only a small percentage (3%) do not believe that ulcerative lesions should be biopsied (answer B). Within its global health promotion program, WHO has created guidelines for oral cancer prevention and control. The document provides the following recommendation: “any lesion that does not significantly improve after 14 days from removal of possible irritant agents should be considered potentially malignant and subjected to biopsy and histological examination” [36]. Question 10 concerned a delicate topic: prescription of systemic antibiotics after an oral biopsy. 68% of the participants answered the question correctly (answer C). Indeed, oral tissue biopsy is usually performed in clean-uncontaminated surgical modalities with low risk of developing clinical infection [37]. Nevertheless, ultra-short and short prophylaxis protocols are indicated in populations at risk, such as immunosuppressed patients and in the presence of prostheses, while in healthy patients only in the case of deep biopsies [38, 39].

## 5. Conclusions

Further studies and surveys are needed to better comprehend the real awareness of dental care professionals regarding the highly sensitive topic of appropriateness. Appropriateness in the dental field must be supported by scientific literature and guidelines to guarantee better assistance to patients, starting from daily practice. The drafting of a coherent list of recommendations regarding appropriateness in Dentistry and in Oral Medicine will follow the present survey, which results from observation of the situation in Italy and will also be published on the Italian Slow Medicine website. These recommendations will be addressed to dentists, oral surgeons, general practitioners and patients and will be conceived as a possible, effective contribution to overall care and patient well-being. The specific purpose of these recommendations is to provide scientific-based guidance for the implementation of appropriate clinical care for the prevention and treatment of oral diseases and for the promotion of oral health. This implies the need for increasing diffusion of correct information. The results of this statistical analysis are preliminary since the sample size analyzed is not probabilistic. This can be a good first step to further investigate.

## Figures and Tables

**Table 1:** Frequency distribution of study participants.

Variables		*N* (%)
Sex	Male	124 (63)
	Female	74 (37)

Degree	Medicine and surgery	54 (27)
	Dentistry and prosthetics	144 (73)

Professional activity	Private	43 (21)
	Public and private	154 (78)
	Public	1 (1)

**Table 2:** Joint distribution of sex, specialty, and degree.

Sex	Specialty	Degree	
		Medicine and surgery	Dentistry
F	Oral surgery	0	3
	Dentistry	1	0
	Orthodontics	0	6
	None	10	54

M	Oral surgery	0	3
	Dentistry	17	0
	Orthodontics	0	4
	None	26	74

**Table 3:** Percentage frequency of answer for each item (the correct one is in bold) and of correct/not-correct answers for each items.

	A	B	C	No error	Error
*Item 1*. Prescription of tooth extraction in pregnancy	**71%**	10%	19%	**71%**	29%
*Item 2*. Biopsy in ulcerative lesion	11%	3%	**86%**	**86%**	14%
*Item 3*. Prescription of antibiotics prior to oral surgery	39%	2%	**59%**	**59%**	41%
*Item 4*. Prescription of topical and systemic antifungal drugs	3%	3%	**94%**	**94%**	6%
*Item 5*. Interruption of antiplatelet or anticoagulant drugs prior to dental extraction	13%	**67%**	20%	**67%**	33%
*Item 6*. Diagnosis of periodontal disease	6%	**72%**	22%	**72%**	28%
*Item 7*. Prescription of extraction of lowering molar	8%	**89%**	3%	**89%**	11%
*Item 8*. Prevention on tumors of oral cavity	**53%**	31%	16%	**53%**	47%
*Item 9.* Prescription of X-ray-OPG	8%	44%	**48%**	**48%**	52%
*Item 10*. Prescription of systemic antibiotics	14%	17%	**69%**	**69%**	31%

**Table 4:** Frequency distribution of number of errors for different kind of specialty.

	Modalities	Oral surgery	Dentistry	Orthodontics	None
*Item 1*. Prescription of tooth extraction in pregnancy	0	50%	94%	70%	70%
	1	50%	6%	30%	30%
*Item 2*. Biopsy in ulcerative lesion	0	100%	94%	70%	86%
	1	0%	6%	30%	14%
*Item 3*. Prescription of antibiotics prior to oral surgery	0	83%	67%	40%	59%
	1	17%	33%	60%	41%
*Item 4*. Prescription of topical and systemic antifungal drugs	0	83%	94%	90%	95%
	1	17%	6%	10%	5%
*Item 5.* Interruption of antiplatelet or anticoagulant drugs prior to dental extraction	0	100%	67%	60%	66%
	1	0%	33%	40%	34%
*Item 6*. Diagnosis of periodontal disease when gum is inflamed	0	67%	72%	60%	73%
	1	33%	28%	40%	27%
*Item 7*. Prescription for extraction of lowering molar	0	100%	94%	80%	88%
	1	0%	6%	20%	12%
*Item 8*. Prevention on tumors of oral cavity	0	50%	78%	40%	51%
	1	50%	22%	60%	49%
*Item 9*. Prescription of X-ray-OPG	0	50%	22%	60%	51%
	1	50%	78%	40%	49%
*Item 10*. Prescription of systemic antibiotics	0	100%	78%	60%	67%
	1	0%	22%	40%	33%

**Table 5:** Results of a logistic regression models.

	Estimate	Std. error	*z* value	*p* value
*Item 1*. Prescription of tooth extraction in pregnancy				
Intercept	−2.79	0.56	−4.96	**7.04*E***−**07**
Gender (male)	0.6	0.35	1.72	0.08
Years of birth (>1972)	0.54	0.38	1.43	0.15
Dentistry degree	1.51	0.56	2.69	**0.01**
Item 6. Inflammatory state of gums				
Intercept	0.23	0.42	0.54	0.59
Gender (male)	−0.51	0.34	−1.51	0.13
Years of birth (>1972)	−0.83	0.34	−2.44	**0.01**
Professional activity (private and public)	−0.61	0.37	−1.65	0.1
*Item 7*. Prescription of lowering molar				
Intercept	−2.94	0.46	−6.42	**1.38*E***−**10**
Years of birth (>1972)	1.4	0.53	2.63	**0.01**
*Item 9*. Prescription of X-ray-OPG				
Intercept	0.84	0.33	2.52	**0.01**
Professional activity (private and public)	−0.97	0.37	−2.62	**0.01**
